# A New Linearized Crank-Nicolson Mixed Element Scheme for the Extended Fisher-Kolmogorov Equation

**DOI:** 10.1155/2013/756281

**Published:** 2013-06-22

**Authors:** Jinfeng Wang, Hong Li, Siriguleng He, Wei Gao, Yang Liu

**Affiliations:** ^1^School of Statistics and Mathematics, Inner Mongolia University of Finance and Economics, Hohhot 010070, China; ^2^School of Mathematical Sciences, Inner Mongolia University, Hohhot 010021, China

## Abstract

We present a new mixed finite element method for solving the extended Fisher-Kolmogorov (EFK) equation. We first decompose the EFK equation as the two second-order equations, then deal with a second-order equation employing finite element method, and handle the other second-order equation using a new mixed finite element method. In the new mixed finite element method, the gradient ∇*u* belongs to the weaker (*L* ^2^(Ω))^2^ space taking the place of the classical **H**(div; *Ω*) space. We prove some a priori bounds for the solution for semidiscrete scheme and derive a fully discrete mixed scheme based on a linearized Crank-Nicolson method. At the same time, we get the optimal a priori error estimates in *L* ^2^ and *H* ^1^-norm for both the scalar unknown *u* and the diffusion term *w* = −Δ*u* and a priori error estimates in (*L* ^2^)^2^-norm for its gradient *χ* = ∇*u* for both semi-discrete and fully discrete schemes.

## 1. Introduction

 In recent years, a lot of numerical methods for solving fourth-order partial differential equations have been presented and analyzed by many researchers. In [1–6], authors studied some mixed finite element methods for fourth-order elliptic and parabolic equations. Shi et al. [7], Wang et al. [8, 9], and H. R. Chen and S. C. Chen [10] proposed some nonconforming finite element methods for fourth-order elliptic equation (or biharmonic equation). In [11], some rectangular finite element methods for fourth-order elliptic singular perturbation problems were considered. Hu and Shi [12] studied the best *L* ^2^ norm error estimate of lower order finite element methods for the fourth-order problem. Chen and Wang [13] discussed a mixed finite element method for thin film epitaxy. In [14], a conforming finite element approximation for the fourth-order Steklov eigenvalue problem is discussed. In [15], a Crank-Nicolson time-stepping was used to approximate the differential term and the product trapezoidal method was employed to treat the integral term, and the quasi-wavelets numerical method for space discretization. Some numerical methods were proposed and studied for Cahn-Hilliard equations, such as (mixed) finite element methods [16–18], time-stepping methods [19, 20], spectral Galerkin method [21], discontinuous Galerkin method [22], and a conservative nonlinear difference scheme [23]. Liu et al. [24] studied a *C* ^1^-conforming finite element method for a fourth-order nonlinear hyperbolic equation. He et al. [25], proposed some mixed element schemes for fourth-order linear wave equation. In [26–28], some (mixed) finite element methods were studied for the extended Fisher-Kolmogorov equations. 

 In [29, 30], authors proposed and discussed a new mixed finite element scheme based on new mixed spaces for second-order linear elliptic equations. In the new mixed method, a weaker (*L* ^2^(*Ω*))^2^ space was provided to replace the complex **H**(div⁡; *Ω*) space. Considering the advantage of the new mixed method, some problems have been studied, such as second-order Sobolev equation [31], second-order parabolic equations [32, 33], and reaction-convection-diffusion problems [34, 35]. However, the new mixed element method for the extended Fisher-Kolmogorov equation has not been studied in the literatures. 

 In this paper, our aim is to apply the new mixed method [29, 30] to solve the extended Fisher-Kolmogorov (EFK) equation [28]

(1) $$\begin{array}{c} u_{t}+\gamma \Delta^{2}u-\Delta u+f\left(u\right)=0,\;\left(x,t\right)\in \Omega \times J, \end{array}$$

with initial condition

(2) $$\begin{array}{c} u\left(x,0\right)=u_{0}\left(x\right),\;x\in \Omega , \end{array}$$

and Dirichlet boundary conditions

(3) $$\begin{array}{c} u\left(x,t\right)=\Delta u\left(x,t\right)=0,\;\left(x,t\right)\in \partial \Omega \times \overset{-}{J}, \end{array}$$

where *Ω* is a bounded convex polygonal domain in *R* ^*d*^, *d* ≤ 2 with boundary ∂*Ω*, and *J* = (0, *T*] is the time interval with 0 < *T* < *∞*. *u*_0_(**x**) is given function, *f*(*u*) = *u* ^3^ − *u* coefficient *γ* is a positive constant. 

 For formulating the new mixed scheme, we first introduce a diffusion term *w* = −Δ*u* to get the following two lower order equations:

(4a) $$\begin{array}{c} u_{t}-\gamma \Delta w+w=-f\left(u\right),\;\left(x,t\right)\in \Omega \times J, \end{array}$$

(4b) $$\begin{array}{c} -\Delta u=w,\;\left(x,t\right)\in \Omega \times J. \end{array}$$

Then, we introduce another auxiliary variable **χ** = ∇*u* in (4b) to obtain the following lower-order system:

(5a) $$\begin{array}{c} u_{t}-\gamma \Delta w+w=-f\left(u\right), \end{array}$$

(5b) $$\begin{array}{c} -\nabla \cdot \chi =w, \end{array}$$

(5c) $$\begin{array}{c} \chi -\nabla u=0. \end{array}$$

For the lower-order system (5a), (5b), and (5c) we will formulate a new mixed scheme based on [29, 30] and discuss some a priori bounds for the mixed element solution and a priori error estimates for semidiscrete scheme. What is more, we will get the fully discrete scheme based on linearized Crank-Nicolson method and some a priori error estimates. From the obtained results, we can find easily that a priori error estimates in *L* ^2^ and *H* ^1^-norm for the scalar unknown *u* and the diffusion term *w* are optimal. Moreover, we also prove a priori error estimate in (*L* ^2^)^2^-norm for the gradient term **χ**. 

 The remainder of this paper is organized as follows. In Section 2, a new mixed weak formulation and semidiscrete mixed scheme are formulated for system (5a), (5b), and (5c), then, some a priori bounds for the new mixed element solution are derived. In Section 3, a new mixed elliptic projection operator 𝒬_*h*_ and a projection operator *𝒮*_*h*_ associated with the coupled systems are presented and some a priori error estimates for semidiscrete scheme are proved. In Section 4, some a priori error estimates for fully discrete linearized Crank-Nicolson scheme are analyzed. Finally in Section 5, some concluding remarks and extensions about the new mixed finite element method are given. In this paper, *C* > 0 is a generic constant which does not depend on the spatial mesh parameter *h* or time step parameter Δ*t*. At the same time, we denote the natural inner product in *L* ^2^(*Ω*) or (*L* ^2^(*Ω*))^2^ by (·, ·) with the corresponding norm ||·||. The others notations and definitions of Sobolev spaces as in [36, 37] are used. In order to facilitate the expression, we denote *B* = *B*(*Ω*), **F** = (*F*(*Ω*))^*d*^, such as *H*_0_^1^≜*H*_0_^1^(*Ω*), **L** ^2^≜(*L* ^2^(*Ω*))^2^, **H** ^1^≜(*H* ^1^(*Ω*))^2^. 

## 2. New Mixed Weak Formulation and Semidiscrete Scheme

 In the following analysis, we will discuss the new mixed formulation and some a priori error estimates for semidiscrete scheme. Based on the lower-order system (5a), (5b), and (5c) the new mixed weak formulation is to find {*u*, *w*, **χ**}:[0, *T*] ↦ *H*_0_^1^ × *H*_0_^1^ × **L** ^2^ such that

(6a) (ut,v)+γ(∇w,∇v)+(w,v)+(f(u),v)=0,∀v∈H01,

(6b) $$\begin{array}{c} \left(\chi ,\nabla g\right)=\left(w,g\right),\;\forall g\in H_{0}^{1}, \end{array}$$

(6c) $$\begin{array}{c} \left(\chi ,z\right)-\left(\nabla u,z\right)=0,\;\forall z\in L^{2}. \end{array}$$

Let *H*_*h*_ and **L**_*h*_, respectively, be finite dimensional subspaces of *H*_0_^1^ and **L** ^2^ defined by the finite element pair *P*_1_ − *P*_0_^2^ [29, 30]

(7) $$\begin{array}{c} H_{h}=\left\{v_{h}\in C^{0}\left(\Omega \right)\cap H_{0}^{1}\;|\;v_{h}\in P_{1}\left(K\right),\forall K\in {𝒦}_{h}\right\},\\ L_{h}=\left\{w_{h}=\left(w_{1h},w_{2h}\right)\in L^{2}\;|\;w_{1h},w_{2h}\in P_{0}\left(K\right),\forall K\in {𝒦}_{h}\right\}. \end{array}$$

Then, the semidiscrete mixed scheme for (6a), (6b), and (6c) is to determine {*u*_*h*_, *w*_*h*_, **χ**_*h*_}:[0, *T*] ↦ *H*_*h*_ × *H*_*h*_ × **L**_*h*_ such that

(8a) (uht,vh)+γ(∇wh,∇vh)+(wh,vh)+(f(uh),vh)=0,∀vh∈Hh,

(8b) $$\begin{array}{c} \left(\chi_{h},\nabla g_{h}\right)=\left(w_{h},g_{h}\right),\;\forall g_{h}\in H_{h}, \end{array}$$

(8c) $$\begin{array}{c} \left(\chi_{h},z_{h}\right)-\left(\nabla u_{h},z_{h}\right)=0,\;\forall z_{h}\in L_{h}. \end{array}$$

In the following discussion, we derive some a priori bounds for the finite element solution.

**Theorem 1**

Let {*u*_*h*_, *w*_*h*_, **χ**_*h*_} be the solution for system (8a), (8b), and (8c) then there exists a constant *C* > 0 such that, for 1 ≤ *p* < +*∞*

(9) ||uh||Lp+||uh||1+||wh||+||χh||+(∫0t||uht||2ds)1/2  ≤C(γ,||u0h||,||w0h||).

**Proof**

Differentiating (8b) and (8c) with respect to *t*, we obtain

(10a) (uht,vh)+γ(∇wh,∇vh)+(wh,vh)+(f(uh),vh)=0,∀vh∈Hh,

(10b) $$\begin{array}{c} -\left(\chi_{ht},\nabla g_{h}\right)+\left(w_{ht},g_{h}\right),\;\forall g_{h}\in H_{h}, \end{array}$$

(10c) $$\begin{array}{c} \left(\chi_{ht},z_{h}\right)-\left(\nabla u_{ht},z_{h}\right)=0,\;\forall z_{h}\in L_{h}. \end{array}$$

Take *v*_*h*_ = *u*_*ht*_, *g*_*h*_ = *w*_*h*_, **z**_*h*_ = ∇*w*_*h*_ in (10a), (10b), and (10c) to obtain

(11a) $$\begin{array}{c} ||u_{ht}{||}^{2}+\gamma \left(\nabla w_{h},\nabla u_{ht}\right)+\left(w_{h},u_{ht}\right)+\left(f\left(u_{h}\right),u_{ht}\right)=0, \end{array}$$

(11b) $$\begin{array}{c} -\gamma \left(\chi_{ht},\nabla w_{h}\right)+\frac{\gamma }{2}\frac{d}{dt}{||w_{h}||}^{2}=0, \end{array}$$

(11c) $$\begin{array}{c} \gamma \left(\chi_{ht},\nabla w_{h}\right)-\gamma \left(\nabla u_{ht},\nabla w_{h}\right)=0. \end{array}$$

We define *F*(*σ*)≜(1/4)(1−*σ* ^2^)^2^ and add the three equations for system (11a), (11b), and (11c) to get

(12) ddt(γ2||wh||2+(F(uh),1))+||uht||2  =−(wh,uht)≤12||wh||2+12||uht||2.

Integrate (12) with respect to time from 0 to *t* and use Gronwall lemma to have

(13) γ2||wh||2+(F(uh),1)+∫0t||uht||2ds  ≤C(γ||wh(0)||2+(F(uh(0)),1)).

We note that *F*(*u*_*h*_) ≥ 0 to obtain

(14) $$\begin{array}{c} ||u_{h}||+||w_{h}||\leq C\left(\gamma ,||u_{0h}||,||w_{0h}||\right). \end{array}$$

Take *g*_*h*_ = *u*_*h*_ in (8b) and **z**_*h*_ = ∇*u*_*h*_ in (8c) to obtain

(15) $$\begin{array}{c} ||\nabla u_{h}||\leq C\left(||u_{h}||+||w_{h}||\right). \end{array}$$

Substitute (14) into (15) to obtain

(16) $$\begin{array}{c} ||\nabla u_{h}||\leq C\left(\gamma ,||u_{0h}||,||w_{0h}||\right). \end{array}$$

Choose **z**_*h*_ = **χ**_*h*_ in (8c) and use (16) to obtain

(17) $$\begin{array}{c} ||\chi_{h}||\leq ||\nabla u_{h}||\leq C\left(\gamma ,||u_{0h}||,||w_{0h}||\right). \end{array}$$

For *Ω* ⊂ *R* ^2^, we use Sobolev imbedding theorem to get

(18) $$\begin{array}{c} {||u_{h}||}_{L^{p}}\leq {||u_{h}||}_{1}\leq C\left(\gamma ,||u_{0h}||,||w_{0h}||\right). \end{array}$$

By (14), (16)–(18), we accomplish the proof of Theorem 1.

**Remark 2**

Based on the discussion in [29, 30], we can see easily that the discrete LBB condition for the mixed finite element space (*H*_*h*_, **L**_*h*_) is satisfied. In our weak formulation (6a), (6b), and (6c) the **H**(div⁡; *Ω*) space is replaced by the weaker **L** ^2^ space. Compared to the **H**(div⁡; *Ω*) space, the regularity requirement for our **L** ^2^ space is reduced.

## 3. A Priori Error Estimates for Semidiscrete Scheme

 In order to analyze the convergence of the method, we first introduce the projection operator *𝒮*_*h*_ and a new mixed elliptic projection operator 𝒬_*h*_ associated with the coupled equations.

**Lemma 3**

Let *𝒮*_*h*_ : [0, *T*] → *H*_0_^1^ → *H*_*h*_ be satisfied by the following relation:

(19) $$\begin{array}{c} \left(\nabla \left(w-{𝒮}_{h}w\right),\nabla v_{h}\right)=0,\;\forall v_{h}\in H_{h},\\ {||w-{𝒮}_{h}w||}_{L^{2}(\Omega )}+h{||w-{𝒮}_{h}w||}_{H^{1}}\leq Ch^{2}{||w||}_{H^{2}}. \end{array}$$

**Lemma 4**

Let (𝒬_*h*_ *u*, 𝒬_*h*_ **χ**):[0, *T*] → *H*_*h*_ × **L**_*h*_ be given by the following new mixed relations:

(20a) $$\begin{array}{c} -\left(\chi -{𝒬}_{h}\chi ,\nabla g_{h}\right)=0,\;\forall g_{h}\in H_{h}, \end{array}$$

(20b) $$\begin{array}{c} \left(\chi -{𝒬}_{h}\chi ,z_{h}\right)-\left(\nabla \left(u-{𝒬}_{h}u\right),z_{h}\right)=0,\;\forall z_{h}\in L_{h}; \end{array}$$

there exists a constant *C* > 0 independent of *h* such that

(21) ||u−𝒬hu||+h(||χ−𝒬hχ||+||u−𝒬hu||1)  ≤Ch2(||u||H2+||χ||H1),||ut−𝒬hut||≤Ch2(||ut||H2+||χt||H1),

from [29, 30], we can obtain the proof for Lemma 4. 

 Further, assuming that the triangulation is quasi-uniform, the following error estimates [27] hold, for *j* = 0,1 and *p* = 2, *∞*:

(22) $$\begin{array}{c} {||{𝒬}_{h}u||}_{W^{j,p}}\leq {||\tau ||}_{W^{j,p}}+{||u||}_{W^{j,p}}\leq C_{u}. \end{array}$$

Based on Lemmas 3 and 4, the following theorem for semidiscrete error estimates is obtained.

**Theorem 5**

With *u*_*h*_(0) = 𝒬_*h*_ *u*_0_, assume that the solution's regular properties for system (6a), (6b), and (6c) satisfy *w*, *u*, *u*_*t*_ ∈ *L* ^*∞*^(*H* ^2^), **χ**, **χ**_*t*_ ∈ *L* ^*∞*^(**H** ^1^). Then there exists a positive constant *C* > 0 independent of *h* such that

(23) ||u−uh||+||w−wh||+h(||u−uh||1+||χ−χh||)  ≤Ch2[||u||L∞(H2)+||χ||L∞(H1)       +(∫0t(||u||H22+||ut||H22+||χ||H12         +||χt||H12+||w||H22)ds)1/2].

**Proof**

To get a priori error estimates, we decompose the errors as

(24) $$\begin{array}{c} w-w_{h}=w-{𝒮}_{h}w+{𝒮}_{h}w-w_{h}=\pi +\kappa ;\\ u-u_{h}=u-{𝒬}_{h}u+{𝒬}_{h}u-u_{h}=\tau +\vartheta ;\\ \chi -\chi_{h}=\chi -{𝒬}_{h}\chi +{𝒬}_{h}\chi -\chi_{h}=\lambda +\zeta . \end{array}$$

Combining (6a)–(8c), (19) and (20a) and (20b), we can get the error equations

(25a) (ϑt,vh)+γ(∇κ,∇vh)+(κ,vh)   +(f(u)−f(uh),vh)  =−(τt,vh)−(π,vh), ∀vh∈Hh,

(25b) $$\begin{array}{c} -\left(\zeta ,\nabla g_{h}\right)+\left(\kappa ,g_{h}\right)=-\left(\pi ,g_{h}\right),\;\forall g_{h}\in H_{h}, \end{array}$$

(25c) $$\begin{array}{c} \left(\zeta ,z_{h}\right)-\left(\nabla \vartheta ,z_{h}\right)=0,\;\forall z_{h}\in L_{h}. \end{array}$$

Differentiating (25b) and (25c) with respect to *t*, we get

(26a) (ϑt,vh)+γ(∇κ,∇vh)+(κ,vh)+(f(u)−f(uh),vh)  =−(τt,vh)−(π,vh), ∀vh∈Hh,

(26b) $$\begin{array}{c} -\left(\zeta_{t},\nabla g_{h}\right)+\left(\kappa_{t},g_{h}\right)=-\left(\pi_{t},g_{h}\right),\;\forall g_{h}\in H_{h}, \end{array}$$

(26c) $$\begin{array}{c} \left(\zeta_{t},z_{h}\right)-\left(\nabla \vartheta_{t},z_{h}\right)=0,\;\forall z_{h}\in L_{h}. \end{array}$$

Choose *v*_*h*_ = *ϑ*_*t*_, *g*_*h*_ = *κ*, and **z**_*h*_ = ∇*κ* in (26a), (26b), and (26c), respectively, to obtain

(27a) ||ϑt||2+γ(∇κ,∇ϑt)+(κ,ϑt)  +(f(u)−f(uh),ϑt)=−(τt,ϑt)−(π,ϑt),

(27b) $$\begin{array}{c} \frac{1}{2}\frac{d}{dt}{||\kappa ||}^{2}-\left(\zeta_{t},\nabla \kappa \right)=-\left(\pi_{t},\kappa \right), \end{array}$$

(27c) $$\begin{array}{c} \left(\zeta_{t},\nabla \kappa \right)-\left(\nabla \vartheta_{t},\nabla \kappa \right)=0. \end{array}$$

Using (27a), (27b), and (27c) and Cauchy-Schwarz inequality and Young inequality, we have

(28) ||ϑt||2+(f(u)−f(uh),ϑt)  =−γ(∇κ,∇ϑt)−(κ,ϑt)−(τt,ϑt)−(π,ϑt)  =−γ(ζt,∇κ)−(κ,ϑt)−(τt,ϑt)−(π,ϑt)  =−γ2ddt||κ||2−(πt,κ)−(κ,ϑt)−(τt,ϑt)−(π,ϑt)  ≤−γ2ddt||κ||2+C(||πt||2+||τt||2+||π||2)   +||κ||2+14||ϑt||2.

Using Cauchy-Schwarz inequality, Young inequality, Sobolev imbedding theorem, and inequality (22), we use the similar method as the one in [27] to obtain

(29) −(f(u)−f(uh),ϑt) =−(f(u)−f(𝒬hu),ϑt)−(f(𝒬hu)−f(uh),ϑt) =−(f(u)−f(𝒬hu),ϑt)−(f(ϑ),ϑt)−3(uh𝒬huϑ,ϑt) ≤|(τ(u2+u𝒬hu+(𝒬hu)2−1),ϑt)|−ddt(F(ϑ),1)  −3(uh𝒬huϑ,ϑt)≤C(||τ||2+||ϑ||2)  −ddt(F(ϑ),1)+14||ϑt||2.

Substituting (29) into (28), we have

(30) ddt[γ2||κ||2+(F(ϑ),1)]+12||ϑt||2 ≤C(||τt||2+||τ||2+||π||2+||πt||2+||ϑ||2+||κ||2).

Integrate (30) with respect to time from 0 to *t* to obtain

(31) γ2||κ||2+(F(ϑ),1)+12∫0t||ϑt||2ds ≤C∫0t(||τt||2+||τ||2+||π||2+||πt||2+||ϑ||2+||κ||2)ds.

Using Gronwall lemma, we have

(32) γ2||κ||2+||ϑ||2+12∫0t||ϑt||2ds ≤C∫0t(||τt||2+||τ||2+||π||2+||πt||2)ds.

Taking *g*_*h*_ = *ϑ* in (25b) and **z**_*h*_ = ∇*ϑ* in (25c), we have

(33a) $$\begin{array}{c} \left(\zeta ,\nabla \vartheta \right)-\left(\kappa ,\vartheta \right)=\left(\pi ,\vartheta \right), \end{array}$$

(33b) $$\begin{array}{c} {||\nabla \vartheta ||}^{2}-\left(\zeta ,\nabla \vartheta \right)=0. \end{array}$$

Add the two equations to get

(34) $$\begin{array}{c} {||\nabla \vartheta ||}^{2}\leq C\left({||\kappa ||}^{2}+{||\vartheta ||}^{2}+{||\pi ||}^{2}\right). \end{array}$$

Choose **z**_*h*_ = **ζ** in (25c) to obtain

(35) ||ζ||2≤||∇ϑ||2≤C(||κ||2+||ϑ||2+||π||2).

Substitute (32) into (34) and (35) to get

(36) ||ζ||2+||∇ϑ||2 ≤C(||π||2+∫0t(||τt||2+||τ||2+||π||2+||πt||2)ds).

Combining Lemmas 3 and 4, (32)–(36) with the triangle inequality, we obtain the error estimates for Theorem 5.

**Remark 6**

The conclusion for Theorem 5 demonstrates that the optimal convergence order in *L* ^2^-norm for both the scalar unknown *u* and the diffusion term *w* is obtained. At the same time, the optimal convergence order in *H* ^1^-norm for the scalar unknown *u* is gotten, too.

**Theorem 7**

Assume that the solution's regular properties for system (6a), (6b), and (6c) satisfy *u*, *w*, *u*_*t*_ ∈ *L* ^*∞*^(*H* ^2^) and **χ**, **χ**_*t*_ ∈ *L* ^*∞*^(**H** ^1^). Then there exists a positive constant *C* independent of *h* such that

(37) ||w−wh||1≤Ch[||u||L∞(H2)+||w||L∞(H2)+||χ||L∞(H1)+(∫0t(||u||H22+||ut||H22+||χ||H12+||χt||H12+||w||H22)ds)1/2].

**Proof**

Choose *v*_*h*_ = *κ*_*t*_, *g*_*h*_ = *ϑ*_*t*_, and **z**_*h*_ = **ζ**_*t*_ in (26a), (26b), and (26c), respectively, to obtain

(38a) (ϑt,κt)+γ2ddt||κ||12+(f(u)−f(uh),κt) =−(τt,κt)−(π,κt),

(38b) $$\begin{array}{c} \left(\zeta_{t},\nabla \vartheta_{t}\right)-\left(\kappa_{t},\vartheta_{t}\right)=\left(\pi_{\text{t}},\vartheta_{t}\right), \end{array}$$

(38c) $$\begin{array}{c} {||\zeta_{t}||}^{2}-\left(\nabla \vartheta_{t},\zeta_{t}\right)=0. \end{array}$$

Adding the three equations of system (38a), (38b), and (38c), we have

(39) γ2ddt||κ||12+||ζt||2 =−(f(u)−f(uh),κt)−(τt,κt)−(π,κt)+(πt,ϑt) =−ddt(f(u)−f(uh),κ)+(f′(u)ut−f′(uh)uht,κ)  −ddt(τt,κ)+(τtt,κ)−ddt(π,κ)+(πt,κ)+(πt,ϑt).

Integrate (39) with respect to *t* and use Cauchy-Schwarz inequality and Young inequality to get

(40) γ2||κ||12+∫0t||ζt||2ds  =−(f(u)−f(uh),κ)   +∫0t(f′(u)ut−f′(uh)uht,κ)ds   −(τt,κ)+∫0t(τtt,κ)ds−(π,κ)+∫0t(πt,κ)ds   +∫0t(πt,ϑt)ds≤−(f(u)−f(uh),κ)   +∫0t(f′(u)ut−f′(uh)uht,κ)ds,C[||τt||2+||π||2+∫0t(||τtt||2+||πt||2+||κ||2+||ϑt||2)ds]   +γ8||κ||2.

Using the similar method to the estimate for (29), we obtain

(41) −(f(u)−f(uh),κ) =−(f(u)−f(Rhu),κ)−(f(Rhu)−f(uh),κ) ≤|(τ(u2+uRhu+(Rhu)2−1),κ)|  +(f′(u^^h)(Rhu−uh),κ) =|(τ(u2+uRhu+(Rhu)2−1),κ)|  +((3(u^^h)2−1)ϑ,κ) ≤C(||τ||2+||ϑ||2)+γ8||κ||12,∫0t(f′(u)ut−f′(uh)uht,κ)ds =∫0t(f′(u)(ut−uht)+(f′(u)−f′(uh))uht,κ)ds =∫0t(f′(u)(ϑt+τt)+f′′(u1)(ϑ+τ)uht,κ)ds ≤C∫0t(||τ||2+||ϑ||2+||τt||2+||ϑt||2+||κ||12)ds.

Substitute (41) into (40) to get

(42) γ4||κ||12+∫0t||ζt||2ds ≤C[||τt||2+||π||2+∫0t(||τ||2+||τt||2+||τtt||2+||πt||2+||κ||12+||ϑ||2+||ϑt||2)ds].

Substitute (32) into (42) and use Gronwall lemma to get

(43) γ4||κ||12+∫0t||ζt||2ds ≤C[||τt||2+||π||2+∫0t(||τ||2+||τt||2+||τtt||2+||π||2+||πt||2)ds].

Combining Lemmas 3 and 4, (43) with the triangle inequality, we obtain the error estimates for Theorem 7.

**Remark 8**

In Theorem 7, the optimal convergence order in *H* ^1^-norm for the diffusion term *w* is obtained.

## 4. A Priori Error Estimates Based on Linearized C-N Scheme 

### 4.1. Linearized Crank-Nicolson Mixed Scheme

 In the following discussion, we will derive the fully discrete a priori error estimates based on a linearized Crank-Nicolson method. Let 0 = *t*_0_ < *t*_1_ < *t*_2_ < ⋯<*t*_*M*_ = *T* be a given partition of the time interval [0, *T*] with step length Δ*t* = *T*/*M* and nodes *t*_*n*_ = *n*Δ*t*, for some positive integer *M*. For a smooth function *ϕ* on [0, *T*], define *ψ* ^*n*^ = *ϕ*(*t*_*n*_) and *ψ* ^*n*−1/2^ = (*ψ* ^*n*^ + *ψ* ^*n*−1^)/2. 

 Equations (6a), (6b), and (6c) can be written as the following formulation at time *t* = *t*_*n*−1/2_:

(44a) (ut(tn−1/2),v)+γ(∇w(tn−1/2),∇v)  +(w(tn−1/2),v)+(f(u(tn−1/2)),v)=0,∀v∈H01,

(44b) $$\begin{array}{c} \left(\chi \left(t_{n-1/2}\right),\nabla g\right)-\left(w\left(t_{n-1/2}\right),g\right)=0,\;\forall g\in H_{0}^{1}m, \end{array}$$

(44c) $$\begin{array}{c} \left(\chi \left(t_{n-1/2}\right),z\right)-\left(\nabla u\left(t_{n-1/2}\right),z\right)=0,\;\forall z\in L^{2}. \end{array}$$

Then, the following equivalent formulation for (44a), (44b), and (44c) is as follows:

(45a) (un−un−1Δt,v)+γ(∇wn−1/2,∇v)   +(wn−1/2,v)+(f(u^^n−1/2),v)  =(R1n−1/2,v)+(R2n−1/2,v), ∀v∈H01,

(45b) $$\begin{array}{c} \left(\chi^{n-1/2},\nabla g\right)-\left(w^{n-1/2},g\right)=0,\;\forall g\in H_{0}^{1}, \end{array}$$

(45c) $$\begin{array}{c} \left(\chi^{n-1/2},z\right)-\left(\nabla u^{n-1/2},z\right)=0,\;\forall z\in L^{2}, \end{array}$$

where

(46) $$\begin{array}{c} {\hat{\hat{u}}}^{n-1/2}=\frac{3}{2}u^{n-1}-\frac{1}{2}u^{n-2},\\ R_{1}^{n-1/2}=\frac{u^{n}-u^{n-1}}{\Delta t}-u_{t}\left(t_{n-1/2}\right),\\ R_{2}^{n-1/2}=f\left({\hat{\hat{u}}}^{n-1/2}\right)-f\left(u\left(t_{n-1/2}\right)\right). \end{array}$$

Now a linearized C-N fully discrete procedure is to find (*u*_*h*_^*n*^, *w*_*h*_^*n*^, **χ**_*h*_^*n*^) ∈ *H*_*h*_ × *H*_*h*_ × **L**_*h*_, (*n* = 0,1,…, *M*) such that

(47a) (uhn−uhn−1Δt,vh)+γ(∇whn−1/2,∇vh)  +(whn−1/2,vh)+(f(u^^hn−1/2),vh)=0, ∀vh∈Hh,

(47b) $$\begin{array}{c} \left(\chi_{h}^{n-1/2},\nabla g_{h}\right)-\left(w_{h}^{n-1/2},g_{h}\right)=0,\;\forall g_{h}\in H_{h}, \end{array}$$

(47c) $$\begin{array}{c} \left(\chi_{h}^{n-1/2},z_{h}\right)-\left(\nabla u_{h}^{n-1/2},z_{h}\right)=0,\;\forall z_{h}\in L_{h}. \end{array}$$

**Remark 9**

We can find that the system (47a), (47b), and (47c) is a linear scheme by a linearized term

(48) $$\begin{array}{c} f\left({\hat{\hat{u}}}_{h}^{n-1/2}\right)={\left(\frac{3}{2}u_{h}^{n-1}-\frac{1}{2}u_{h}^{n-2}\right)}^{3}-\left(\frac{3}{2}u_{h}^{n-1}-\frac{1}{2}u_{h}^{n-2}\right). \end{array}$$

In the following subsection, we derive a priori error estimates based on a linearized Crank-Nicolson fully discrete scheme. 

### 4.2. A Priori Error Estimates for Fully Discrete Scheme

 In order to derive the linearized C-N fully discrete error estimates, we now write the errors as follows:

(49) $$\begin{array}{c} w\left(t_{n}\right)-w_{h}^{n}=w\left(t_{n}\right)-{𝒮}_{h}w^{n}+{𝒮}_{h}w^{n}-w_{h}^{n}=\pi^{n}+\kappa^{n};\\ u\left(t_{n}\right)-u_{h}^{n}=u\left(t_{n}\right)-{𝒬}_{h}u^{n}+{𝒬}_{h}u^{n}-u_{h}^{n}=\tau^{n}+\vartheta^{n};\\ \chi \left(t_{n}\right)-\chi_{h}^{n}=\chi \left(t_{n}\right)-{𝒬}_{h}\chi^{n}+{𝒬}_{h}\chi^{n}-\chi_{h}^{n}=\lambda^{n}+\zeta^{n}. \end{array}$$

Subtracting (47a), (47b), and (47c) from (45a), (45b), and (45c) and using (19) and (20a) and (20b) at *t* = *t*_*n*−1/2_, we have the following error equations

(50a) (ϑn−ϑn−1Δt,vh)+γ(∇κn−1/2,∇vh)+(κn−1/2,vh)  +(f(u^^n−1/2)−f(u^^hn−1/2),vh)=−(τn−τn−1Δt,vh)  −(πn−1/2,vh)+(R1n−1/2,vh)+(R2n−1/2,vh), ∀vh∈Hh,

(50b) (ζn−1/2,∇gh)−(κn−1/2,gh)=(πn−1/2,gh),∀gh∈Hh,

(50c) $$\begin{array}{c} \left(\zeta^{n-1/2},z_{h}\right)-\left(\nabla \vartheta^{n-1/2},z_{h}\right)=0,\;\forall z_{h}\in L_{h}. \end{array}$$

In order to get the fully discrete error estimates, we introduce the following lemma.

**Lemma 10**

For *R*_1_^*n*−1/2^ and *R*_2_^*n*−1/2^, the following estimates hold:

(51) $$\begin{array}{c} ||R_{1}^{n-1/2}||\leq C\Delta t^{2}{||u_{ttt}||}_{L^{\infty }(L^{2})},\\ ||R_{2}^{n-1/2}||\leq C\left(u\right)\Delta t^{2}{||u_{ttt}||}_{L^{\infty }(L^{2})}. \end{array}$$

**Proof**

Using the Taylor expansion, we can obtain easily the conclusion for Lemma 10.

**Remark 11**

From the estimate for ||*R*_2_^*n*−1/2^|| in Lemma 10, we know that the convergence rate in time direction for the system (47a), (47b), and (47c) is order 2, so the system (47a), (47b), and (47c) is called a linearized Crank-Nicolson scheme based on the linearized term $f\left({\hat{\hat{u}}}_{h}^{n-1/2}\right)$.

In the following discussion, we will derive some fully discrete a priori error estimates.

**Theorem 12**

With *u*_*h*_^0^ = 𝒬_*h*_ *u* ^0^, assume that the solution's regular properties for system (6a), (6b), and (6c) satisfy *u*_*t*_ ∈ *L* ^2^(*H* ^2^), *u*_*ttt*_ ∈ *L* ^*∞*^(*L* ^2^), *u*, *w* ∈ *L* ^*∞*^(*H* ^2^), **χ** ∈ *L* ^*∞*^(**H** ^1^), **χ**_*t*_ ∈ *L* ^2^(**H** ^1^). Then there exists a positive constant *C* independent of *h* and Δ*t* such that

(52) ||u(tJ)−uhJ||≤C[h2|||·|||+Δt2||uttt||L∞(L2)],

|||·|||≜||*u*||_*L*^*∞*^(*H*^2^)_ + ||*u*_*t*_||_*L*^2^(*H*^2^)_ + ||*w*||_*L*^*∞*^(*H*^2^)_ + ||**χ**||_*L*^*∞*^(**H**^1^)_ + ||**χ**_*t*_||_*L*^2^(**H**^1^)_.

**Proof**

Setting *v*_*h*_ = *ϑ* ^*n*−1/2^, *g*_*h*_ = *κ* ^*n*−1/2^, and **z**_*h*_ = ∇*κ* ^*n*−1/2^ in (50a), (50b), and (50c), respectively, we get

(53a) ||ϑn||2−||ϑn−1||22Δt+γ(∇κn−1/2,∇ϑn−1/2)  +(κn−1/2,ϑn−1/2)+(f(u^^n−1/2)−f(u^^hn−1/2),ϑn−1/2) =−(τn−τn−1Δt,ϑn−1/2)−(πn−1/2,ϑn−1/2)  +(R1n−1/2,ϑn−1/2)+(R2n−1/2,ϑn−1/2),

(53b) $$\begin{array}{c} \gamma {||\kappa^{n-1/2}||}^{2}-\gamma \left(\zeta^{n-1/2},\nabla \kappa^{n-1/2}\right)=-\gamma \left(\pi^{n-1/2},\kappa^{n-1/2}\right), \end{array}$$

(53c) $$\begin{array}{c} \gamma \left(\zeta^{n-1/2},\nabla \kappa^{n-1/2}\right)-\gamma \left(\nabla \vartheta^{n-1/2},\nabla \kappa^{n-1/2}\right)=0. \end{array}$$

Adding the three equations of (53a), (53b), and (53c) and using Cauchy-Schwarz inequality and Young inequality, we have

(54) γ||κn−1/2||2+12Δt(||ϑn||2−||ϑn−1||2) =−(κn−1/2,ϑn−1/2)−(f(u^^n−1/2)−f(u^^hn−1/2),ϑn−1/2)  −(τn−τn−1Δt,ϑn−1/2)  −(πn−1/2,ϑn−1/2)+(R1n−1/2,ϑn−1/2)+(R2n−1/2,ϑn−1/2)  −γ(πn−1/2,κn−1/2)≤(||τn−τn−1Δt||+||R1n−1/2||+||R2n−1/2||)||ϑn−1/2||  +||κn−1/2||||ϑn−1/2||+γ||πn−1/2||||κn−1/2||  +(||f(u^^n−1/2)−f(u^^hn−1/2)||+||πn−1/2||)||ϑn−1/2||≤C(||τn−τn−1Δt||2+||R1n−1/2||2+||R2n−1/2||2+||πn−1/2||2) +γ2||κn−1/2||2+C||f(u^^n−1/2)−f(u^^hn−1/2)||2 +C||ϑn−1/2||2.

In order to accomplish our process, we have to estimate the nonlinear term ||f(u^^n-1/2)-f(u^^hn-1/2)||2. Using Young inequality, we have

(55) ||f(u^^n−1/2)−f(u^^hn−1/2)||2≤2||(u^^n−1/2)3−(u^^hn−1/2)3||2+2||u^^n−1/2−u^^hn−1/2||2=2||[32(un−1−uhn−1)−12(un−2−uhn−2)]   ×[(32un−1−12un−2)2+(32uhn−1−12uhn−2)2     +(32un−1−12un−2)(32uhn−1−12uhn−2)]||2  +2||32(un−1−uhn−1)−12(un−2−uhn−2)||2≤2||32(ϑn−1+τn−1)−12(ϑn−2+τn−2)||2 ×[||(32un−1−12un−2)2+(32uhn−1−12uhn−2)2    +(32un−1−12un−2)(32uhn−1−12uhn−2)||L∞2+1] =(24Cu2+2)||32(ϑn−1+τn−1)−12(ϑn−2+τn−2)||2≤C(||τn−1||2+||τn−2||2+||ϑn−1||2+||ϑn−2||2).

Note that

(56) $$\begin{array}{c} {||\frac{\tau^{n}-\tau^{n-1}}{\Delta t}||}^{2}\leq \frac{1}{\Delta t}\int_{t_{n-1}}^{t_{n}}{||\tau_{t}(s)||}^{2}ds. \end{array}$$

Substitute (55) and (56) into (54) to obtain

(57) γ2||κn−1/2||2+12Δt(||ϑn||2−||ϑn−1||2)≤C(1Δt∫tn−1tn||τt(s)||2ds+||R1n−1/2||2    +||R2n−1/2||2+||πn−1/2||2) +C(||τn−1||2+||τn−2||2+||ϑn||2+||ϑn−1||2+||ϑn−2||2).

Summing from *n* = 2,…, *J*, the resulting inequality becomes

(58) γΔt∑n=2J||κn−1/2||2+(1−CΔt)||ϑJ||2≤||ϑ1||2+CΔt∑n=2J(||R1n−1/2||2+||R2n−1/2||2+||πn−1/2||2) +CΔt∑n=0J−1(||τn||2+||ϑn||2)+C∫0tJ||τt||2ds.

For sufficiently small Δ*t*, using Gronwall lemma with Lemma 10, we get

(59) γΔt∑n=2J||κn−1/2||2+12||ϑJ||2 ≤||ϑ1||2+CΔt∑n=0J−1||τn||2  +CΔt∑n=2J(||R1n−1/2||2+||R2n−1/2||2+||πn−1/2||2)  +C∫0tJ||τt||2ds≤||ϑ1||2  +C(||τn||L∞(L2)2+||πn−1/2||L∞(L2)2      +∫0tJ||τt||2ds)+CΔt4||uttt||L∞(L2)2.

Use Lemmas 3 and 4, (59) with the triangle inequality to accomplish the proof of Theorem 12.

**Theorem 13**

With *u*_*h*_^0^ = 𝒬_*h*_ *u* ^0^, assume that the solution's regular properties for system (6a), (6b), and (6c) satisfy *u*_*t*_ ∈ *L* ^2^(*H* ^2^), *u*_*ttt*_ ∈ *L* ^*∞*^(*L* ^2^), *u*, *w* ∈ *L* ^*∞*^(*H* ^2^), **χ** ∈ *L* ^*∞*^(**H** ^1^), **χ**_*t*_ ∈ *L* ^2^(**H** ^1^). Then there exists a positive constant *C* independent of *h* and Δ*t* such that

(60a) $$\begin{array}{c} {||u\left(t_{J}\right)-u_{h}^{J}||}_{1}\leq C\left[h||\left|\cdot \right|||+\Delta t^{2}{||u_{ttt}||}_{L^{\infty }(L^{2})}\right], \end{array}$$

(60b) (Δt∑n=1J||w(tn−1/2)−whn−1/2||j2)1/2 ≤C[h2−j|||·|||+Δt2||uttt||L∞(L2)], j=0,1,

(60c) $$\begin{array}{c} ||\chi \left(t_{n-1/2}\right)-\chi_{h}^{n-1/2}||\leq C\left[h||\left|\cdot \right|||+\Delta t^{2}{||u_{ttt}||}_{L^{\infty }(L^{2})}\right]. \end{array}$$

**Proof**

Choose *v*_*h*_ = *κ* ^*n*−1/2^, *g*_*h*_ = (*ϑ* ^*n*^ − *ϑ* ^*n*−1^)/Δ*t*, and **z**_*h*_ = (∇*ϑ* ^*n*^ − ∇*ϑ* ^*n*−1^)/Δ*t* in (50a), (50b), and (50c) to obtain

(61a) (ϑn−ϑn−1Δt,κn−1/2)+γ||∇κn−1/2||2+||κn−1/2||2  +(f(u^^n−1/2)−f(u^^hn−1/2),κn−1/2) =−(τn−τn−1Δt,κn−1/2)−(πn−1/2,κn−1/2)  +(R1n−1/2,κn−1/2)+(R2n−1/2,κn−1/2),

(61b) (ζn−1/2,∇ϑn−∇ϑn−1Δt)−(κn−1/2,ϑn−ϑn−1Δt) =(πn−1/2,ϑn−ϑn−1Δt),

(61c) $$\begin{array}{c} -\left(\zeta^{n-1/2},\frac{\nabla \vartheta^{n}-\nabla \vartheta^{n-1}}{\Delta t}\right)+\frac{{||\nabla \vartheta^{n}||}^{2}-{||\nabla \vartheta^{n-1}||}^{2}}{2\Delta t}=0. \end{array}$$

We add the above three equations of (61a), (61b), and (61c) to get

(62) ||∇ϑn||2−||∇ϑn−1||22Δt+γ||∇κn−1/2||2+||κn−1/2||2 =−(f(u^^n−1/2)−f(u^^hn−1/2),κn−1/2)  −(τn−τn−1Δt,κn−1/2)−(πn−1/2,κn−1/2)  +(R1n−1/2,κn−1/2)+(R2n−1/2,κn−1/2)  +(πn−1/2,ϑn−ϑn−1Δt) =−(f(u^^n−1/2)−f(u^^hn−1/2),κn−1/2)  −(τn−τn−1Δt,κn−1/2)−(πn−1/2,κn−1/2)  +(R1n−1/2,κn−1/2)+(R2n−1/2,κn−1/2)  +(πn,ϑn)−(πn−1,ϑn−1)Δt−(ϑn−1/2,πn−πn−1Δt).

For (62), we employ Cauchy-Schwarz inequality as well as Young inequality to obtain

(63) ||∇ϑn||2−||∇ϑn−1||22Δt+γ||∇κn−1/2||2+12||κn−1/2||2≤C(||f(u^^n−1/2)−f(u^^hn−1/2)||2+||τn−τn−1Δt||2   +||πn−1/2||2+||R1n−1/2||2+||R2n−1/2||2   +||ϑn−1/2||2+||πn−πn−1Δt||2) +(πn,ϑn)−(πn−1,ϑn−1)Δt.

Multiply (63) by 2Δ*t* and then sum from *n* = 2,…, *J* to get

(64) ||∇ϑJ||2+2γΔt∑n=2J||∇κn−1/2||2 +Δt∑n=2J||κn−1/2||2≤||∇ϑ1||2 +CΔt∑n=2J(||f(u^^n−1/2)−f(u^^hn−1/2)||2       +||τn−τn−1Δt||2+||πn−1/2||2       +||R1n−1/2||2+||R2n−1/2||2       +||ϑn−1/2||2+||πn−πn−1Δt||2) +(πJ,ϑJ)−(π1,ϑ1).

Substituting the following inequality

(65) $$\begin{array}{c} {||\frac{\pi^{n}-\pi^{n-1}}{\Delta t}||}^{2}\leq \frac{1}{\Delta t}\int_{t_{n-1}}^{t_{n}}{||\pi_{t}(s)||}^{2}ds, \end{array}$$

and (55) into (64), we obtain

(66) ||∇ϑJ||2+2γΔt∑n=2J||∇κn−1/2||2+Δt∑n=2J||κn−1/2||2 ≤||ϑ1||12+||πJ||2  +CΔt∑n=2J(||τn−1||2+||τn−2||2+||ϑn−1||2        +||ϑn−2||2+||πn−1/2||2+||R1n−1/2||2        +||R2n−1/2||2+||ϑn−1/2||2)+||ϑJ||2.

Substitute (59) into (66) to obtain

(67) ||∇ϑJ||2+2γΔt∑n=2J||∇κn−1/2||2 +Δt∑n=2J||κn−1/2||2≤||ϑ1||12 +CΔt∑n=2J(||τn−1||2+||τn−2||2+||πn−1/2||2       +||R1n−1/2||2+||R2n−1/2||2)+||πJ||2 +C(||τn||L∞(L2)2+∫0tJ||τt||2ds     +Δt4||uttt||L∞(L2)+||πn−1/2||L∞(L2)2).

Taking **z**_*h*_ = **ζ** ^*n*−1/2^ in (50c), we have

(68) $$\begin{array}{c} {||\zeta^{n-1/2}||}^{2}\leq C{||\nabla \vartheta^{n-1/2}||}^{2}\leq C\left({||\vartheta^{n}||}^{2}+{||\vartheta^{n-1}||}^{2}\right). \end{array}$$

Combining Lemmas 3–10, (67), and (68) with the triangle inequality, we accomplish the proof.

## 5. Some Concluding Remarks and Extensions

 In [29, 30], authors proposed a new mixed finite element method, which has been applied to solve second-order evolution equations, such as Sobolev equations [31], parabolic equations [32, 33], and reaction-convection-diffusion problems [34, 35]. In this paper, we apply the new mixed finite element scheme [29, 30] to solve the extended Fisher-Kolmogorov (EFK) equation (fourth-order nonlinear reaction diffusion equation). Compared to the classical mixed methods, the weaker square integrable **L** ^2^ space, which takes the place of the complex **H**(div⁡; *Ω*), is used in our method. We derive some a priori bounds for the solution and a priori error estimates for semidiscrete scheme. What's more, we obtain a priori error estimates for fully discrete scheme by a linearized Crank-Nicolson method. 

 If we take *γ* = 0 in (1), we can get the following second-order nonlinear reaction-diffusion equation:

(69) $$\begin{array}{c} u_{t}-\Delta u+f\left(u\right)=0,\;\left(x,t\right)\in \Omega \times J, \end{array}$$

with initial condition

(70) $$\begin{array}{c} u\left(x,0\right)=u_{0}\left(x\right),\;x\in \Omega , \end{array}$$

and Dirichlet boundary conditions

(71) $$\begin{array}{c} u\left(x,t\right)=0,\;\left(x,t\right)\in \partial \Omega \times \overset{-}{J}, \end{array}$$

where *Ω* is a bounded convex polygonal domain in *R* ^*d*^, *d* ≤ 2 with boundary ∂*Ω*, and *J* = (0, *T*] is the time interval with 0 < *T* < *∞*. *u*_0_(**x**) is given function, *f*(*u*) = *u* ^3^ − *u*. 

 Using a similar method as the one in this paper, we can get the following weak formulation for system (69)–(71):

(72a) $$\begin{array}{c} \left(u_{t},v\right)+\left(\lambda ,\nabla v\right)+\left(f\left(u\right),v\right)=0,\;\forall v\in H_{0}^{1}, \end{array}$$

(72b) $$\begin{array}{c} \left(\lambda ,z\right)-\left(\nabla u,z\right)=0,\;\forall z\in L^{2}, \end{array}$$

where ***λ*** = ∇*u*. Based on the mixed weak formulation (72a) and (72b), we can get the similar theoretical analysis as our method in this paper. 

 In the future work, we will apply the new mixed scheme to solve fourth-order linear/nonlinear wave equations [24, 25, 38]. At the same time, we will study the large time-stepping method based on the new mixed element scheme for the Cahn-Hilliard equation [19, 20]. 
